# System-wide analysis of qualitative hospital incident data: Feasibility of semi-automated content analysis to uncover insights

**DOI:** 10.1177/18333583241299433

**Published:** 2024-11-23

**Authors:** Teyl Engstrom, Danelle Kenny, Wallace Grimmett, Mary-Anne Ramis, Chris Foley, Clair Sullivan, Jason D. Pole

**Affiliations:** 1 The University of Queensland, Australia; 2 Mater Health, Australia; 3 Royal Brisbane and Women’s Hospital, Australia; 4 The University of Toronto, Canada

**Keywords:** routinely collected health data, patient safety, risk management, qualitative research, hospital, quality data reporting, health information management

## Abstract

**Background:**

Advances in technology have increased the ease of reporting hospital incidents, resulting in large amounts of qualitative descriptive data. Health services have little experience analysing these data at scale to incorporate into routine reporting.

**Objective:**

We aimed to explore the feasibility of applying a semi-automated content analysis (SACA) tool (Leximancer™) to qualitative descriptions of system-wide hospital incidents to provide insights into safety issues at all health service levels.

**Method:**

Data from 1245 incidents reported across a network of hospitals in Australia were analysed using the SACA tool. Summaries were generated using a variety of techniques, including inductive and deductive approaches to extract key concepts in the data.

**Results:**

The analysis was feasible and provided an actionable summary of the types of incidents reported in the data; the visual interface allowed users to explore the underlying text for a deeper understanding. Deductive analysis was utilised to explore specific areas of interest, and stratified analysis revealed more detailed concepts. The SACA tool was more efficient than manual processes; however, due to the context present in the incident descriptions, significant time, reading and subject matter expertise is still required to refine the analysis.

**Conclusion:**

Semi-automated tools provide an opportunity for improving patient safety culture and practices by providing rapid content analysis of vast datasets that can be customised for specific organisational contexts and deployed at scale. Further research is required to assess usefulness with system users.

**Implications:**

Qualitative data abound and system-wide analysis is essential to creating actionable insights.

## Introduction

Healthcare systems are committed to the delivery of high-quality health care, including the maintenance of quality standards in care provision (Australian Commission on Safety and Quality in Health Care, 2022; Canadian Patient Safety Institute, 2020; National Board of Health and Welfare, 2020; The Joint Commission, 2022; World Health Organisation, 2019). There is an expectation that healthcare systems will enable a high standard of care that is timely, safe, and accessible (Australian Commission on Safety and Quality in Health Care, 2022). To maintain public trust, health services are required to adhere to national safety and quality standards, which formulate the accreditation and reporting requirements for providing health services in their jurisdiction, such as those set in Australia (Australian Commission on Safety and Quality in Health Care, 2022), North America (Canadian Patient Safety Institute, 2020; The Joint Commission, 2022) and Sweden (National Board of Health and Welfare, 2020). Patient safety is a component of these standards and has been embedded in clinical governance frameworks in Australia, and other jurisdictions, as a high priority since the 1990s (Clay-Williams et al., 2017).

Despite the prioritisation of patient safety and the oversight of clinical governance frameworks, incidents still frequently occur in hospitals (Morello et al., 2013). An incident is defined as ‘an event or circumstance that resulted, or could have resulted, in unintended or unnecessary harm to a patient or consumer; or a complaint, loss or damage’ and includes a near miss which is ‘an incident or potential incident that was averted and did not cause harm, but had the potential to do so’ (Australian Commission on Safety and Quality in Health Care, 2022). Incidents occur due to the complex, dynamic nature of care delivery, the diversity of roles, perspectives and beliefs of staff, and the difficulties faced by diverse organisations in reaching consensus on definitions of safety culture (Moon et al., 2022; Morello et al., 2013). Developing a safety culture requires mechanisms for reporting, knowledge generation and learning from incidents (Morello et al., 2013; Pacenko et al., 2024).

With the increasing availability of digital technology, health services are implementing software to collect data pertaining to safety incidents (Queensland Government Department of Health, 2014). Such solutions provide fast and easy data collection and a convenient way for individuals to report incidents (Leary et al., 2020). Qualitative incident descriptions are favoured as they allow users to more accurately capture the incident and its evolution (Health Quality Ontario, 2017). However, as more qualitative data are generated it becomes increasingly complex and time-consuming to analyse (World Health Organisation, 2020), especially when considering incidents across entire health systems. As more important details are included, the more subtle nuances provided by these details can be lost or obscured by the vast amounts of data (Ismail et al., 2020).

Incident management guidelines recommend that health services assign sufficient resources and training to analyse and investigate recorded incidents (Australian Commission on Safety and Quality in Health Care, 2021). While health services invest human resources to analyse the information, these tasks are often in addition to their standard role, to be analysed in ‘spare time’, and often too voluminous to process within tight time parameters (Krumholz, 2014; Randell et al., 2009). Therefore, analyses of safety incident reports are limited, often based on only frequency, perceived impact or incidents from a single clinical organisation unit. The current manual approach to data analysis is not sustainable. Such approaches are limited and omit large amounts of data from analysis, resulting in missed opportunities to improve system-wide quality care and patient safety (Westbrook et al., 2015).

New tools capable of analysing large volumes of qualitative data are required as part of an integrated incident management systems to perform the ‘Analyser’ role as defined by Runciman et al. (2006). Semi-automated content analysis (SACA) tools provide an option to perform both content frequency and relational analysis in an efficient manner. These tools utilise statistical algorithms and machine learning identify patterns and present visualisations and summaries that represent the significance of the concepts within the text and their relationships (Smith and Humphreys, 2006). The purpose of this research was to explore the feasibility of using a SACA tool to analyse qualitative hospital incident descriptions to produce a fast and useful synthesis of the data across a health service in Queensland, Australia.

## Method

### Setting and data

This was a retrospective analysis of routinely collected qualitative safety incident descriptions from a network of hospitals operated by a not-for-profit organisation in South-East Queensland, Australia. These hospitals offer tertiary level care across all clinical domains with approximately 1500 beds. When an incident occurs, hospital policy requires any staff member involved to record details in the incident management system. Most of the incident record is entered as free-text, with some drop-down selections available for categorising details such as setting, incident type and subsequent action or escalation.

After Ethical and Governance approvals, data were extracted from the incident management system by the clinical governance team, into an Excel file. Identifying details, including patient or staff names or numbers were removed and replaced them with a system generated identifier. Data included both clinical and non-clinical incidents from the single month of April 2021, totaling 1245 incidents. The dataset included a column with the qualitative description of the incident, which was the focus of the qualitative data analysis. The dataset also contained several variables relating to the incident, including the location, date and severity assessment code (SAC).

#### Ethics approval

This study was granted ethics approval through the Mater Misericordiae Ltd. Human Research Ethics Committee (EC00332, HREC/MML/80145 (V2)) and registered with The University of Queensland Human Research Ethics Office.

### Leximancer™ semi-automated content analysis

Leximancer™ is a SACA tool that uses machine learning to extract prevalent concepts and visually display their connectivity (Smith and Humphreys, 2006). Leximancer can efficiently capture insights hidden in vast amounts of qualitative data (Leximancer Pty Ltd, 2021; Smith and Humphreys, 2006) and has been validated in several studies (Engstrom et al., 2022; MacCarthy and Shan, 2022; Smith and Humphreys, 2006). In addition to the visual display, users can view the raw text tagged with each concept, enabling them to easily read the relevant text and understand more about the concepts.

The process of running text through Leximancer is highly automated; once the text is uploaded, it is possible to generate a summary within minutes (Leximancer Pty Ltd., 2021). However, there are many settings which can be configured to produce a more nuanced output. Leximancer visually represents concepts and themes and their connectivity in a concept map (Leximancer Pty Ltd, 2021). Concept maps were first developed in the 1970s as a means to enable conceptual understanding of topics and in recent decades have been digitised through various software programs (Novak and Cañas, 2006).The Leximancer concept map includes small black dots, each representing a concept identified in the text. Concepts are then grouped into themes, which are represented as large, coloured bubbles. The colours represent a heat map of relative importance or prevalence of the theme, with warmer colours (red, orange) being more prevalent and cooler colours (purple, blue) being less prevalent. Concepts that frequently appear together in the text will be displayed closer together in the concept map.

#### Iterative inductive analysis

By default, Leximancer performs an inductive analysis, discovering prevalent concepts based only on the text provided. Concepts are collections of words that co-occur through the text (Leximancer Pty Ltd, 2021). Each term is weighted by frequency, comparing co-occurrence with frequency found elsewhere. Sentence blocks are tagged with a concept when a threshold of accumulated evidence is reached. The software automatically identifies high-frequency concepts and their co-occurrence, using this information to generate an inductive concept map.

Users can remove words with low semantic content within the context of the text, known as ‘stop words’ (Leximancer Pty Ltd, 2021). One example is ‘and’ – this word appears in written English with very high frequency, but when considered in isolation does not contribute to the analysis. Starting with the default English stop-word list, we (DK and TE) reviewed the initial output and found the analysis had identified several non-determinate words as concepts. We added these words to the stop-word list and reran the analysis. This process was repeated until all non-determinate concepts were excluded.

Users can define compound concepts when there are two or more concepts that have a specific meaning when they occur together (Leximancer Pty Ltd, 2021). Concepts can be combined with a combination of ‘AND’, ‘OR’ or ‘NOT’ operators. We (DK and TE) reviewed the concept list and context in which they were used to identify if there was a need to create compound concepts.

#### Deductive analysis

If there is an item of interest or enquiry that is not automatically detected, there is functionality to apply a deductive approach (Leximancer Pty Ltd, 2021). Users can define concepts and, if present in the data, force them to appear on the concept map (called the user-defined concept feature). We (DK and TE) utilised this feature to explore if and how the concept of ‘scrubs’ appeared in the incident description text. This term was chosen based on anecdotal evidence from stakeholders that there may be a related issue.

#### Sub-group analysis

Health services are interested in the association of clinical incidents with level of harm, which can be investigated by sub-group analysis (Deeks et al., 2019). We (DK and TE) investigated whether specific types of incidents were associated with higher levels of harm. Data were stratified by SAC, ascribed to incidents from levels 1 to 4: SAC 4 indicates incidents with *no harm* or a *near miss*; SAC 3 indicates *minimal harm*; SAC 2 indicates *temporary harm*; and SAC 1 indicates *death* or *likely permanent harm* (Queensland Government Department of Health, 2014). We included SAC as a variable and used the Leximancer tagging feature to tag text with the related SAC. We then included SAC 4 as a Kill Concept, which removes those incidents from the analysis, leaving only incidents with a reported SAC 1–3. We then compared the results to the analysis of the full dataset.

## Results

### Iterative inductive analysis

**Figure 1.**
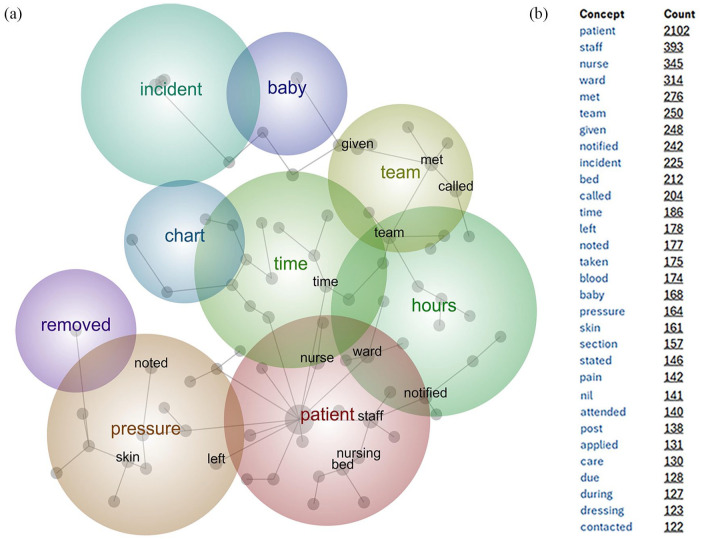
Leximancer™ initial analysis – concept map and top ranked concepts. Figure depicts (a) the initial concept map and (b) top ranked concepts prior to adjusting any settings.

The initial analysis of the incident data returned a set of concepts and themes that identified ‘patient’ as the most frequently occurring concept, related to essentially every other concept. The default concept map and list of ranked concepts is displayed in Figure 1. Other highly prevalent concepts included ‘nurse’, ‘staff’ and ‘ward’.

**Figure 2.**
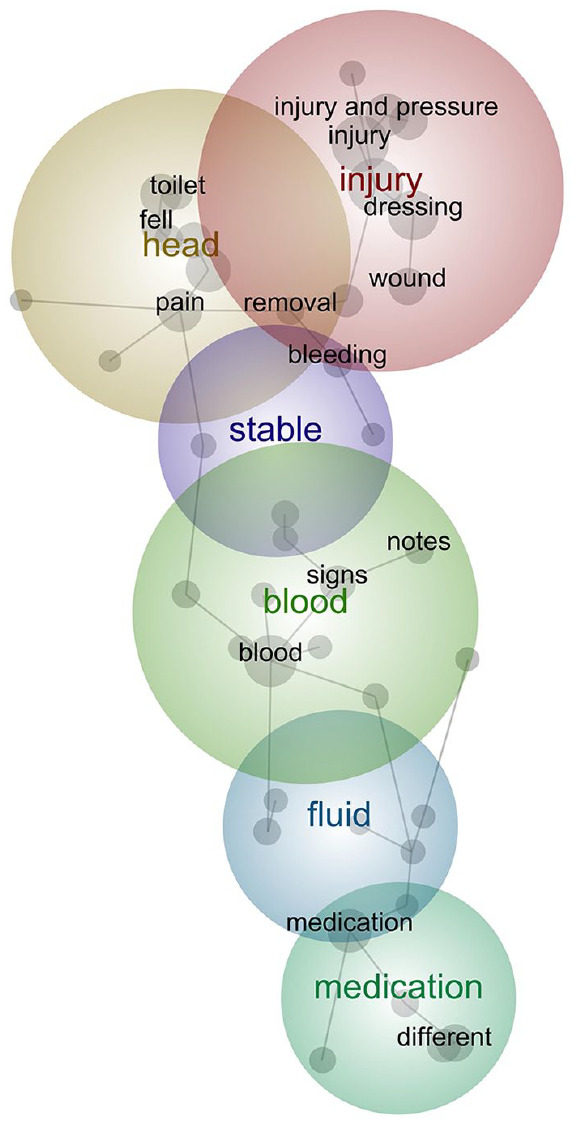
Final inductive analysis concept map. Figure depicts the resulting concept map after removing stop words and utilising compound concepts to separate concepts relating to ‘pressure’ into ‘blood pressure’, ‘pressure injuries’ and other pressure-related concepts.

The purpose of utilising a SACA tool was to provide a useful synthesis of the incidents reported. However, high-frequency non-determinate terms dominated the initial Leximancer analysis. We identified and removed these terms from the analysis by adding them to the stop-word list, producing a refined concept map. This process was applied seven times, with each iteration generating a more focused concept map (Figure 2), and fewer remaining non-determinate terms (Table 1). These terms were identified through querying each concept and looking at the context around the use of the word. If the concept assisted in the identification of patient safety issues, it was retained. Any areas of uncertainty were discussed with the project team until consensus was achieved. Non-determinate language included words that were used in most descriptions (e.g. ‘patient’, ‘nurse’, ‘care’); and words that reflected patterns of writing (e.g. ‘via’, ‘approx.’, ‘having’).

**Table 1.** Iterative inductive analysis stop-words.

Iteration number	Stop-words added
0	Default English stop-word list
1	patient, nurse, staff, ward, incident, notified, given, noted, bed, called, attended, stated, taken, left, care, arrived, section, applied, contacted, required, used, removed, due, room, asked, floor, area, further, received, during, phone, commenced, home, down, prior, noticed, bag, via
2	pt, time, advised, review, team, checked, left, transferred, informed, order, observations, baby, charted, present, completed, dr, day, placed, assessment, nil, shift, admission, aware, admitted, hrs, handover, hand, sent, infusion, hospital, multiple
3	assist, returned, chart, reported, reviewed, check, discharge, request, use, transfer, administered, minutes, morning, doctor, leader, normal, approx., provided, rate, member, told, immediate, obs, previous, plan, place, arm
4	changed, manager, needed, ordered, requested, cleaned, remained, arrival, theatre, form, confirmed, able, family, procedure, birth, information, code, emergency, risk, night, surgeon, treatment, having
5	monitoring, management, change, presented, performed, days, regarding, using, met, today, bedside, support, approximately
6	booked, alert, need, chair, saline, overnight, level, moved, ongoing, despite
7	attempted, take, scan, sitting, handed, checking

There were a large number of stop words identified through this process, as the descriptions often included not just the incident itself but also additional context such as the patient’s movement prior to the incident, or how the incident was responded to. This is expected from a reporting perspective as staff are encouraged to include as much relevant details as possible. However, it creates complexity in isolating the cause of the incident. Due to the complexity of the text, significant reading time and subject matter expertise was required for this process. The stop words identified through this process are likely to be fairly stable over time but would need to be revisited periodically.

In some instances, certain concepts were important to the analysis but, on their own, may have concealed other concepts if included on the concept map. In our data, we found the concept of ‘pressure’ had multiple contexts such as blood pressure and pressure injuries. To account for this, we created compound concepts for (i) ‘pressure’ AND ‘injuries’; (ii) ‘pressure’ AND ‘blood’; and (iii) ‘pressure’ AND NOT (‘blood’ OR ‘injuries’), to capture other uses of pressure. We then included the three compound concepts in the concept map and excluded the overall concept of pressure. By removing the concept from the map but retaining the compound concepts, the two compound concepts were separated.

The final concept map presented in Figure 2 identified ‘injury’ as the most prevalent theme, this included concepts such as ‘pressure injury’, ‘dressing’, ‘wound’, ‘red’, ‘skin’, ‘stage’ and ‘removal’. This significant cause of hospital incidents often included a description of the stage of the pressure injury and how the wounds were treated and dressed. ‘Head’ was the second most prevalent theme and relates to patients or staff hitting their head. Some of these incidents relate to falls, and a common location was identified as the bathroom, which is important for staff to understand to prevent future falls. A theme of ‘medication’ was also identified through the analysis, including concepts of ‘different’, ‘correct’ and ‘incorrect’. This theme describes an issue with medication orders containing multiple different patient labels, or patients being prescribed or issued incorrect medication. Other themes represented in the concept map were ‘blood’, ‘fluid’ and ‘stable’.

### Deductive analysis

**Figure 3.**
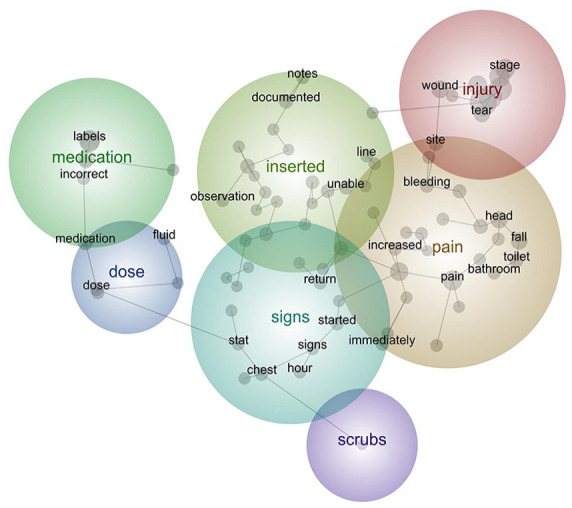
Forced concept map. Figure depicts the concept map from a deductive analysis investigating the concept ‘scrubs’, which was largely unconnected to other issues within the data.

We performed a deductive analysis, forcing the software to include the concept ‘scrubs’ (Figure 3). Four text segments were identified that were tagged with this concept, and co-located with the concept ‘chest’, but generally unrelated to other concepts in the map. The low frequency of this term explains why it was not included as a concept in the inductive analysis. The four incidents tagged with this concept each described different unrelated issues, not providing evidence of a systematic issue as hypothesised by the hospital network. In this example, the clinical governance team were able to investigate possible safety concerns and confirm the absence of an issue based on clinical safety reports. There may be other occasions where staff members raise safety concerns that are confirmed by this process. Therefore, deductive analysis is an efficient tool that can focus on enquiries, facilitating rapid, accurate responses to already identified or emerging patient safety concerns.

### Sub-group analysis

**Figure 4.**
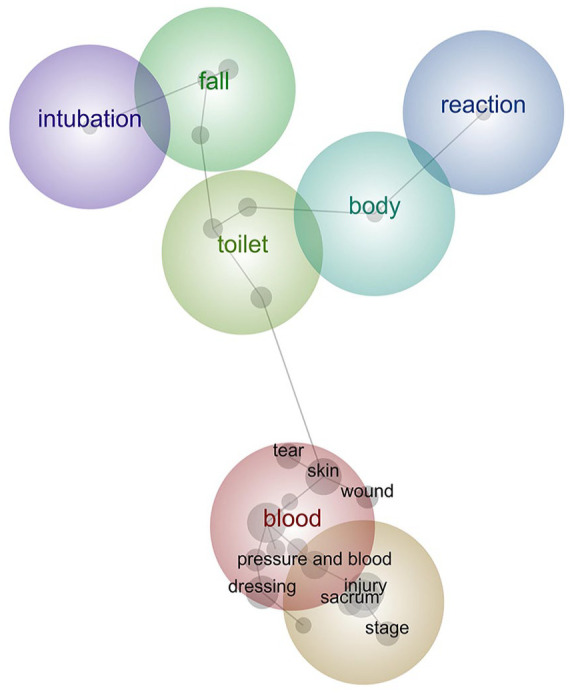
Sub-group SAC 1–3 concept map. Figure depicts the concept map from a sub-group analysis focusing on incidents which caused some harm (SAC level 1–3). SAC: severity assessment code.

Sub-group analysis examined incidents with a SAC of 1–3 only. Of the 1245 incidents in the dataset, 234 (19%) incidents had a SAC 1–3, indicating the incident resulted in some level of harm not reasonably expected as an outcome of healthcare (Queensland Government Department of Health, 2014). We analysed the SAC 1–3 incidents using the same analysis settings used in the final iteration of the inductive analysis based on the full dataset. The concept map (Figure 4) shows that the theme of ‘blood’ became the most prevalent (from third most prevalent in overall analysis), indicating that blood-related incidents were more likely to result in harm. The ‘pressure injuries’ theme was second most prevalent and now included additional concepts, such as ‘sacrum’, revealing more detailed information about the location of these injuries. The theme relating to ‘bathroom’, ‘toilet’ and ‘falls’ in the overall analysis had now split into two. New themes related to ‘intubation’ and ‘reaction’ were identified through this sub-group analysis, demonstrating flexibility and adaptability to different settings in this type of analysis.

## Discussion

Our study found that SACA could process large volumes of system-wide qualitative hospital incident data into an actionable summary that can be queried from multiple perspectives, to deepen understanding of reported incidents. The content analysis provided a direct link from the synthesised concepts to the raw text, to enable a deeper understanding. These tools still require expert user input and time to initially identify stop words and configure the settings appropriately for the context. There is potential for SACA to reduce the administrative burden associated with analysis of patient incident reports and make outputs more accessible for staff to identify opportunities, improve patient safety, as well as be used for governance and reporting.

There is an increasing need to find alternatives to the current methods of reviewing incident reports. The volume of qualitative data captured in incident reporting systems is increasing (Queensland Government Department of Health, 2014; World Health Organisation, 2020; Young et al., 2019), and in most health services, these are read manually by hospital staff or only analysed as part of research projects. Due to the resource and skills required for such efforts, often only the highest impact reports are reviewed for deeper analysis and actions (Krumholz, 2014; Randell et al., 2009). It may take months to review reported incidents, by which time opportunities for improvement are missed or potentially may have progressed from near misses to actual harm (Westbrook et al., 2015). This causes a perception among staff that their qualitative reports are not reviewed nor actioned, increasing the risk of them not reporting future incidents (Hartnell et al., 2012; Varallo et al., 2018). Not having efficient and effective mechanisms to analyse and action incident reports is a risk to maintaining and improving patient safety culture (Health Quality Ontario, 2017). There is a potential of mitigating this risk with the inclusion of SACA as a framework for incident data analysis.

The SACA tool used for this analysis (Leximancer Pty Ltd, 2021; Smith and Humphreys, 2006) is relatively simple to use and adaptable to the needs of the organisation. The automation of the data classification provides objectivity and removes some of the potential human bias inherent in qualitative analysis. However, it should be noted that input from staff with subject matter expertise is required to calibrate the customisable settings, including the stop-word list. This is especially important for the incident description data used in this analysis as the text included a lot of contextual information as well as a description of the incident itself. Significant time was required to finetune the settings, and this may need to be updated in different clinical settings, and may benefit from clinician input (Trinh et al., 2023). While the first pass of the data added little insight, updating the stop words and project settings resulted in a concept map that generated insights that directed the attention of users toward actionable outcomes.

Multiple different levels and types of analyses may be required to cater to different aspects of patient safety within a health service (Meyer et al., 2003). This includes accessing information within both ‘push’ and ‘pull’ frameworks (Kong et al., 2021; Metabase, 2023). By default, the SACA takes an inductive approach, pushing information to users by automatically identifying concepts and weighting them by frequency, accumulating evidence that the particular concept exists (Leximancer Pty Ltd, 2021). This type of analysis may be most useful for high-level clinical governance, where teams want to monitor how the type and frequency of incidents are changing over time or compare across jurisdictions (Zhan et al., 2005). The SACA also enables users to pull information they require through the use of user-defined concepts to explore terms of particular interest (Leximancer Pty Ltd, 2021). These terms may not be identified as a concept by default because their frequency is low (Kong et al., 2021; Randell et al., 2009); however, users can specify a concept and the tool will build a thesaurus around that concept and include it on the map. This approach may be useful for reporting on specific key performance indicators (Sittig et al., 2015); it could also be used to investigate the prevalence and context of specific hospital issues raised through anecdotal evidence.

While demonstrating the validity and reliability of Leximancer was outside of the scope of this study, other studies that have done so. Studies comparing Leximancer to manual thematic analysis reported that both methods generally found similar concepts; however, Leximancer was less likely to identify emotive concepts and concepts that required more contextual interpretation (Engstrom et al., 2022; MacCarthy and Shan, 2022; Penn-Edwards, 2010). Similarly, studies comparing Leximancer to other qualitative analysis software tools such as NVivo reported both tools proved useful in answering the research question, and there was significant overlap in the concepts identified, but Leximancer was limited in identifying contextual and tonal intricacies (Sotiriadou et al., 2014; Wilk et al., 2019). These authors valued Leximancer visual output and speed, but found NVivo better enabled storytelling due to these researchers being closer to the data through the manual data handling required (Sotiriadou et al., 2014; Wilk et al., 2019). While Leximancer was utilised in this study, other SACA tools provided similar functionality.

### Limitations

Our study has some limitations, the most apparent being model scalability – further investigation is required into the operationalisation of this solution. The stop-word list generated was specific to this dataset; generation of the stop-word list and compound concepts needs to be culturally and contextually derived, so refining these settings may need to be done within each setting and may need updating over time. It should be noted that the analysis is dynamic and, hence, the visualisation may change slightly each time it is run. While it does not change the outcome or the story, users should be aware of this. The output requires human interpretation, which can introduce bias. The information on hospital incidents is limited to what was reported in the system, and there may be additional incidents which went unreported, or reported in less detail, resulting in an incomplete view. Despite these limitations, SACA presents an opportunity to harness technology to provide fast, effective insights to improve the quality of patient care and enhance safety culture within our healthcare system.

### Implications

- Increasing amounts of data are being generated through digital incident reporting systems and a lack of resources to analyse these data is a major barrier to improving patient safety.
- SACA software can provide a useful summary of large volumes of qualitative hospital incident data. The analysis method highlighted in this article can provide a more efficient solution compared to current manual approaches to analysing hospital incident data.

## Conclusion

A major barrier to health services utilising incident reporting to drive improved patient safety is a lack of resources to analyse the incident description data and action the findings at scale (Health Quality Ontario, 2017; World Health Organisation, 2020). As increasing amounts of data are generated through digital incident reporting systems, the current manual approaches are ‘swamped’ by the volume of data. Thus, they are not suitable and inevitably result in delayed or missed opportunities to prevent further risks to patient and caregiver safety. The current study demonstrated that it is feasible to use SACA solutions to interrogate qualitative incident data quickly and efficiently in multiple ways, producing customisable summaries. However, due to the volume of context present in the incident descriptions, these analysis techniques do not take the human out of the loop and require time and subject matter expertise to refine the analysis. More research is required to assess the usefulness of this output with system users.
